# A CuNi/C Nanosheet Array Based on a Metal–Organic Framework Derivate as a Supersensitive Non-Enzymatic Glucose Sensor

**DOI:** 10.1007/s40820-017-0178-9

**Published:** 2017-12-22

**Authors:** Li Zhang, Chen Ye, Xu Li, Yaru Ding, Hongbo Liang, Guangyu Zhao, Yan Wang

**Affiliations:** 1 School of Chemistry and Chemical Engineering, Harbin Institute of Technology, Harbin, 150080 Heilongjiang People’s Republic of China; 2 Department of Ophthalmology, Second Hospital, Jilin University, Changchun, 130022 Jilin People’s Republic of China

**Keywords:** Non-enzymatic glucose sensor, Nanoparticle, Nanosheet array, Self-supported electrode, Copper–nickel bimetal catalyst

## Abstract

Bimetal catalysts are good alternatives for non-enzymatic glucose sensors owing to their low cost, high activity, good conductivity, and ease of fabrication. In the present study, a self-supported CuNi/C electrode prepared by electrodepositing Cu nanoparticles on a Ni-based metal–organic framework (MOF) derivate was used as a non-enzymatic glucose sensor. The porous construction and carbon scaffold inherited from the Ni-MOF guarantee good kinetics of the electrode process in electrochemical glucose detection. Furthermore, Cu nanoparticles disturb the array structure of MOF derived films and evidently enhance their electrochemical performances in glucose detection. Electrochemical measurements indicate that the CuNi/C electrode possesses a high sensitivity of 17.12 mA mM^−1^ cm^−2^, a low detection limit of 66.67 nM, and a wider linearity range from 0.20 to 2.72 mM. Additionally, the electrode exhibits good reusability, reproducibility, and stability, thereby catering to the practical use of glucose sensors. Similar values of glucose concentrations in human blood serum samples are detected with our electrode and with the method involving glucose-6-phosphate dehydrogenase; the results further demonstrate the practical feasibility of our electrode.

**Electronic supplementary material:**

The online version of this article (10.1007/s40820-017-0178-9) contains supplementary material, which is available to authorized users.

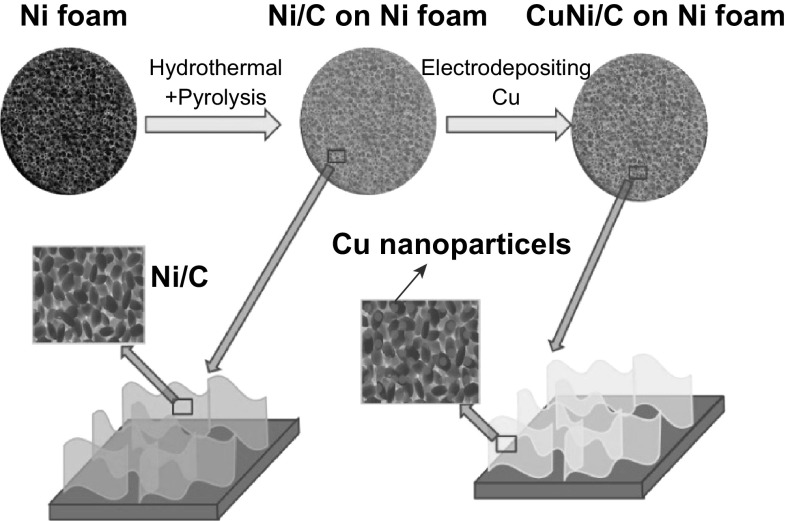

## Highlights

- CuNi/C nanosheet arrays were prepared by pyrolyzing Ni-based metal organic framework and successive Cu electrodeposition.
- The prepared arrays exhibited high sensitivity (17.12 mA mM^−1^ cm^−2^) and low detection limit (66.67 nM) as non-enzymatic glucose sensors.
- The electrode exhibits good reusability, reproducibility, and stability and thereby caters to the practical use of glucose sensors.

## Introduction

Quantitative analysis of glucose in blood is vital for diabetes mellitus diagnosis, and obtaining these values conveniently and accurately is especially desirable [1, 2]. Despite their high sensitivity and selectivity, classical electrochemical enzyme sensors still present disadvantages including high cost, instability, and poor reproducibility. These drawbacks are due to the intrinsic defects of glucose oxidase or glucose dehydrogenase [1–5]. Therefore, there is a tremendous need to develop inexpensive and robust non-enzymatic sensors that can accurately detect glucose in blood. Diverse catalysts, such as carbon composites, noble metals, transition metals, transition metal oxides and hydroxides, metal alloys, and bimetals, were explored as non-enzymatic glucose sensors [6–23]. Among these, transition metals attract considerable attention owing to their low cost, high conductivity, good catalytic activity, and facile preparation [24–41]. In various transition metals, Ni nanocrystals exhibit a series of outstanding performances when they are used as glucose sensors including high sensitivity, low detection limit, and good stability [6, 42, 43]. However, the narrow linearity range of Ni catalysts in glucose detection restricts their practicability [43]. A typical method to solve this issue involves introducing another metal to form a bimetal catalyst that enhances the properties by completely using the components in catalysts [44, 45]. Previous studies [44–46] indicate that Cu metal possesses a significantly wider linearity range in glucose detection when compared with Ni [47, 48], and thus researchers explored several bimetal catalysts by combining the advantages of Cu and Ni with comprehensive virtues. Wang [49] reported a CuNi bimetal catalyst that presents a wide linearity range from 7 μM to 23.67 mM that considerably exceeds those of Ni catalysts. Li et al. [50] indicated that CuNi nanocrystal composite behaved much better than a catalyst with a single component due to the bi-functional effect. Researchers also compared electrochemical properties of carbon nanotubes modified with CuNi to those modified with a single metal. The results demonstrated that bimetals exhibited higher response currents and lower detection limit [51, 52]. In a previous study, we reported a self-supported electrode constructed with porous Ni/C nanosheets derived from Ni-based metal organic frameworks (MOFs) [43]. The electrode exhibited attractive characteristics with respect to glucose detection, owing to its unique hierarchically porous structure, small crystals, and a wide-spread carbon scaffold. However, a narrow linearity range (0.15 μM–1.48 mM) was the only disadvantage of this electrode. In order to compensate for this drawback, in the present study, Cu was introduced into the porous nanosheet arrays electrode to form a self-supported CuNi/C glucose sensor.

Furthermore, MOFs derivates display significant potential when they are used in electrocatalysis and electrochemical energy storage owing to their porous structure, ultra-small active materials, and homogeneously conductive carbon scaffold. Additionally, the homogeneous distribution of pores, active grains, and carbon derived from the unique construction of MOFs enables their derivates to act as good candidates for self-supported electrodes. We can integrate conductive frame, mass transport channels, and nanoscale active materials by using a bottom-up process as opposed to the traditional pasting method for preparing electrodes. In this study, the electrodes were prepared by electrodeposited Cu nanoparticles on the porous Ni/C nanosheet arrays that were prefabricated via pyrolyzing Ni-based MOFs on Ni foam [43]. CuNi/C electrodes inherit the superiority of hierarchically porous structures and good conductivity from Ni/C substrates and thereby enable the CuNi/C glucose sensor to perform with a high sensitivity of 17.12 mA mM^−1^ cm^−2^ and a low detection limit of 66.67 nM. The compensation of Cu nanoparticles causes the CuNi/C electrodes to also exhibit a wider linearity range (0.2 μM–2.72 mM) when compared with the Ni/C electrode.

## Experimental

### Materials

All reagents are analytical reagents, and they were used without further purification. Specifically, CuSO_4_·5H_2_O, Na_2_SO_4_, NiSO_4_·6H_2_O, aqueous ammonia (25–28%), NiCl_2_·6H_2_O, C_8_H_6_O_4_, N, N-dimethylacetamide (DMF), D-(+)-Glucose, dopamine (DA), L-ascorbic acid (AA), acetaminophen, fructose, sucrose, folic acid, and l-cysteine were purchased from Sinopharm Chemical Reagent Co., Ltd (China). Additionally, K_2_S_2_O_8_ was purchased from Tianjin Kaitong Chemical Reagent Co., Ltd. (Tianjin, China). Uric acid (UA) was purchased from Alfa Aesar.

### Preparation of CuNi/C Electrodes

The electrodes were prepared by electrodepositing Cu nanoparticles on the porous Ni/C nanosheet arrays that were prefabricated via pyrolyzing Ni-MOFs on Ni foam [43]. First, 0.11 g NiCl_2_·6H_2_O and 0.08 g C_8_H_6_O_4_ were dissolved in a 25-mL mixture of DMF/ethanol/water (v:v:v = 14:1:1). The solution was mixed by magnetic stirring for 30 min and then poured into a 40-mL Teflon-lined autoclave with pieces of Ni foam (2 × 1 cm^2^). The hydrothermal reaction was performed in an oven at 125 °C for 24 h. The Ni foam was removed after the autoclave naturally cooled to room temperature. The foam was alternately rinsed with absolute ethanol and DI water several times and then dried overnight. Furthermore, Ni/C arrays were obtained by pyrolyzing the Ni-MOF in Ar atmosphere at 420 °C for 4 h with a ramp rate of 2 °C min^−1^. Second, the electrodeposition of Cu nanoparticles was conducted in a three-electrode system with Ni/C arrays as a working electrode by using a Pt foil (1 × 1 cm^2^) as a counter electrode and a saturated calomel electrode as a reference electrode. Moreover, Cu depositing was performed in an electrolyte of 0.1 M CuSO_4_ + 0.2 M Na_2_SO_4_ by using a potentiostatic method at −1.4 V. Finally, the obtained CuNi/C electrodes were rinsed with DI water and ethanol several times and dried overnight for further characterization.

### Physical Characterization and Electrochemical Measurements of the CuNi/C Electrodes

Scanning electron microscope (SEM) images and energy-dispersive spectra (EDS) were obtained on a Hitachi Su-8100 (Japan). The X-ray diffraction (XRD) patterns were obtained on a PANalytical X´pert PRO X-ray diffractometer with Cu Kα radiation (*λ* = 1.54 Å). Raman spectra were recorded from 750 to 3500 cm^−1^ on a Renishaw 2000 Confocal Raman Microprobe (Renishaw Instruments, England) by using a 514.5 nm argon ion laser. Transmission electron microscope (TEM) images were obtained on a JEOL-2100 (Japan). The electrochemical measurements were taken on a CHI 660D workstation (CH Instruments, China). A three-electrode system was used for testing with the CuNi/C as working electrode (apparent area is 1 × 1 cm^2^) by using a Pt foil and an Ag/AgCl electrode as counter and reference electrodes, respectively. All potentials were relative to the Ag/AgCl (sat’d KCl) electrode in 0.1 M NaOH electrolyte. Cyclic voltammetry (CV) was performed in the quiescent solution, and amperometric measurements were taken under magnetic stirring. With respect to the 60-day stability test, our electrode was washed with DI water and dried naturally at room temperature for the next test (after every test at an interval of 5 days).

## Results and Discussion

**Fig. 1:**
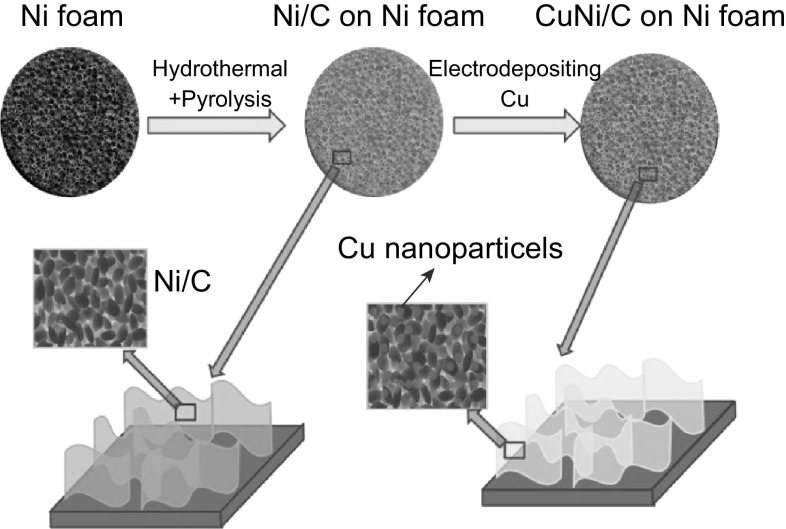
Schematic diagram of preparing CuNi/C electrodes

**Fig. 2:**
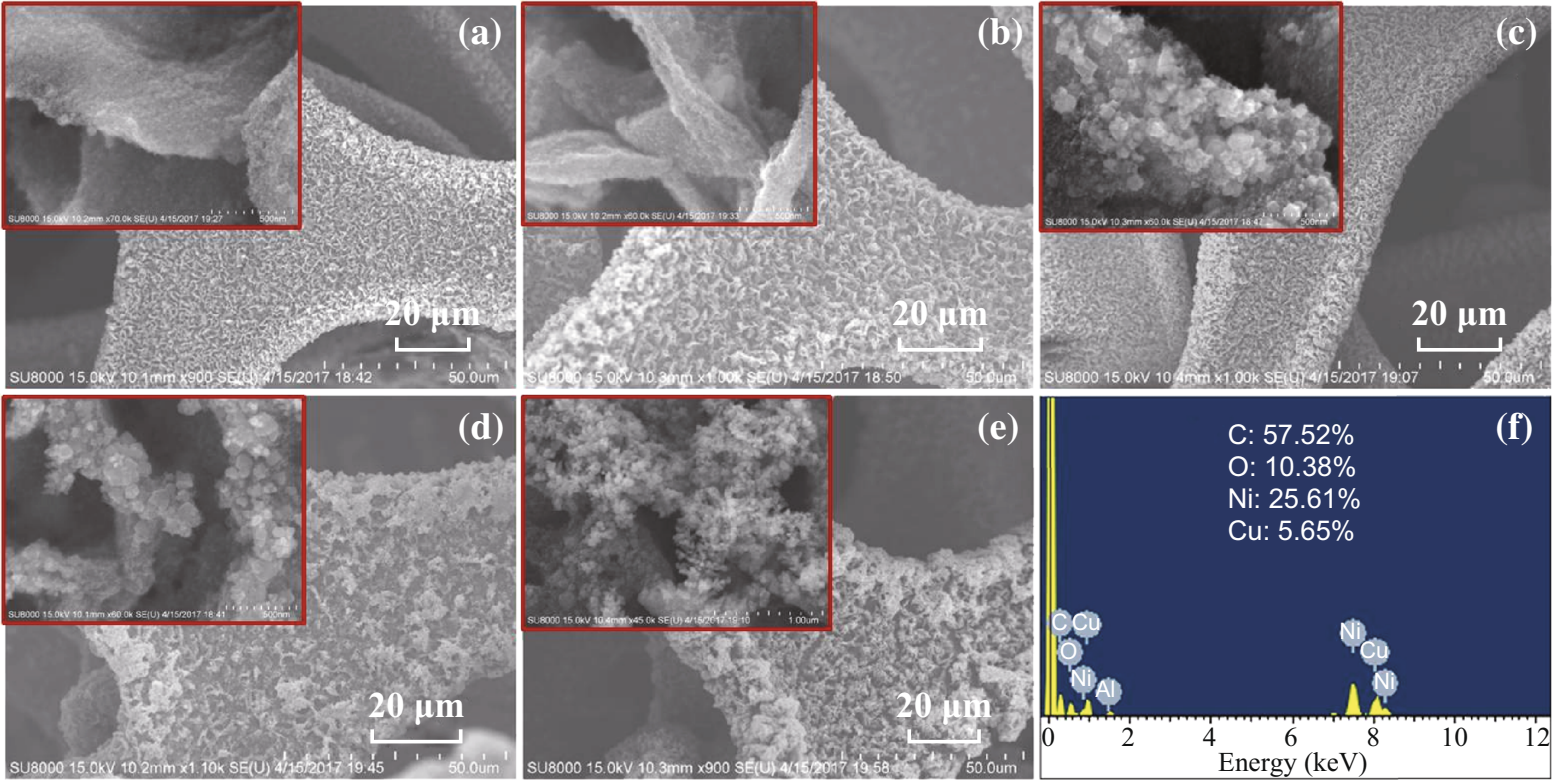
SEM images of CuNi/C electrodes prepared with different Cu depositing times: **a** 5 s, **b** 10 s, **c** 20 s, **d** 50 s, and **e** 100 s. The insets show the corresponding high-resolution images, and **f** EDS pattern on **c**

**Fig. 3:**
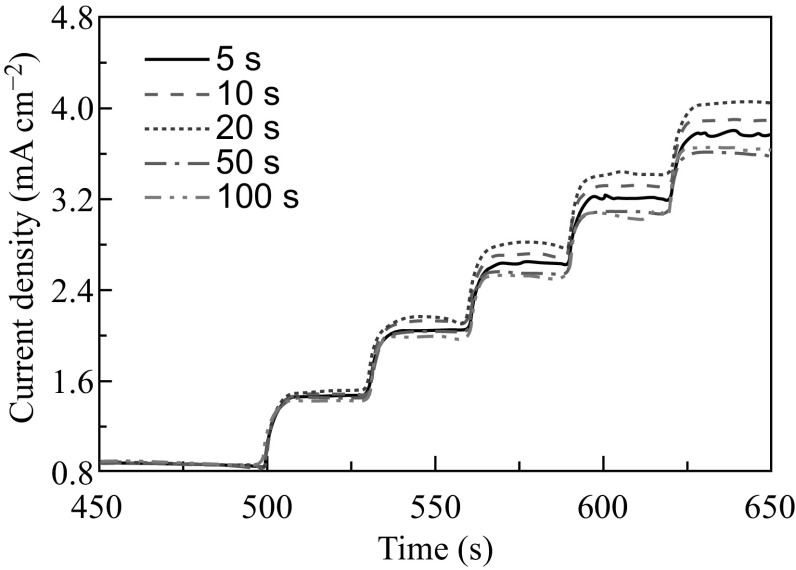
Amperometric responses of CuNi/C obtained from different Cu depositing time, and the detail shows the consecutive addition of 20 μM glucose into 0.1 M NaOH at 0.54 V

The CuNi/C electrodes were prepared according to the schematic diagram shown in Fig. 1. Porous Ni/C nanosheet membranes derived from Ni-terephthalic acid MOFs (Ni_3_(OH)_2_(C_8_H_4_O_4_)_2_(H_2_O)_4_) were successfully anchored on a Ni foam substrate by using hydrothermal treatment with successive pyrolysis [43]. Subsequently, CuNi/C electrodes were prepared by electrodepositing Cu nanoparticles on Ni/C electrodes. The quantity and size of Cu nanoparticles were adjusted by adopting different depositing times. As shown in Fig. S1, the surface morphologies of the samples in which the deposited time exceeds 20 s were compared with the raw Ni/C electrode. As shown in Fig. 2a, b, there is no obvious change when the depositing time is 5 or 10 s, and this indicates that a low amount of Cu is deposited on the Ni/C surface. Conversely, excessive Cu deposition blocks the channels between the nanosheets when the depositing time exceeds 20 s (i.e., 50 or 100 s). This is unfavorable for the mass transport while using the electrodes for glucose detection. The amperometric responses of CuNi/C electrodes with different Cu depositing times describe the relationship between the catalytic ability and the Cu amounts as shown in Fig. 3. Evidently, the sample with Cu depositing time of 20 s exhibits the highest response current, thereby indicating the optimal preparation parameter. As shown in Fig. 2f, with the optimal preparation parameter, the EDS results demonstrate that the atom ratio of C/Ni/Cu is 10:2:1. This indicates that the primary component of CuNi/C arrays is carbon. In contrast, the element mapping (shown in Fig. S2) reveals the homogeneous distribution of C, Cu, and Ni on the electrode. The TEM images of raw Ni/C nanosheets (Fig. S3a, b) exhibit the homogeneous distribution of Ni and C nanoparticles in the nanosheets as described in detail in a previous study [43]. After Cu depositing, larger nanoparticles were detected on the nanosheets heterogeneously (as denoted by red arrows in Fig. S3c, d), and this is consistent with the SEM results. Figure S4 shows a more comprehensive morphology detail of CuNi/C electrode. Evidently, the Cu nanoparticles are homogeneously distributed on the Ni/C nanosheets without interference with respect to the array structure and micrometer channels. This aids in improving the catalytic ability of Ni/C while not disturbing the kinetics in electrochemical detection.

**Fig. 4:**
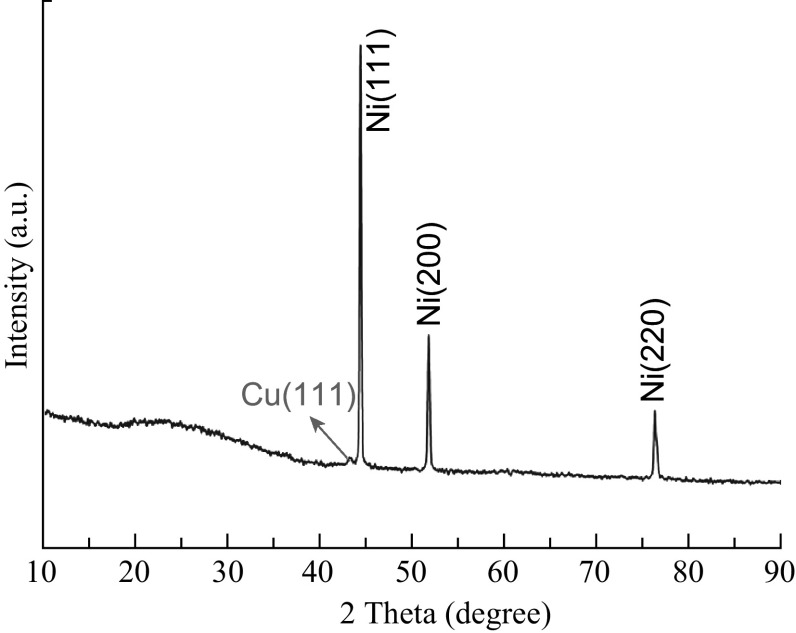
XRD pattern of CuNi/C electrodes

The existence of Cu in the as-prepared electrodes is further confirmed by XRD analyses as shown in Fig. 4. A small peak assigned to Cu(111) (JCPDS card No. 04-0836) is detected at 43.3° with the exception of strong diffraction peaks of Ni from Ni/C substrates and Ni foam. For comparison purposes, the XRD pattern of porous Ni/C on Ni foam is shown in Fig. S5. The results indicate that the pattern of Ni/C on Ni foam primarily exhibits Ni diffraction peaks (JCPDS card No. 04-0850). In order to further exclude the interference of Ni foam, Ni/C powder prepared with the same condition is also measured by XRD analyses. Evidently, the Ni primary content in the powder reveals that the Ni catalyst on electrodes results from the pyrolysis of Ni-MOF. Conversely, the bump at approximately 26° is ascribed to the amorphous carbon from pyrolysis.

**Fig. 5:**
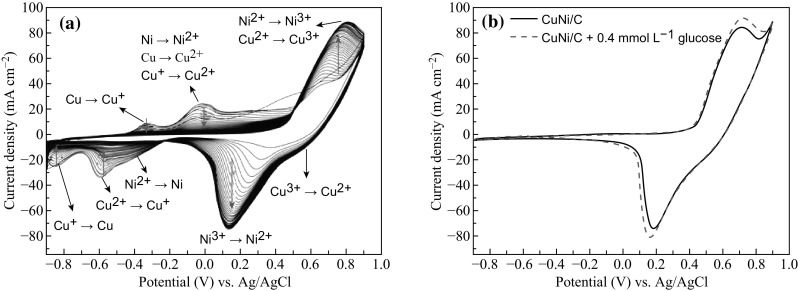
**a** Activated CuNi/C electrodes in 0.1 M NaOH by CV for 60 cycles. **b** CVs of activated electrodes in 0.1 M NaOH and 0.1 M NaOH + 0.4 mM glucose. The CV measurements are performed in a three-electrode system by using Pt foil as counter electrode, and Ag/AgCl as a reference electrode. The scan rate is 20 mV s^−1^

The CuNi/C electrodes were activated by CV ranging from − 0.9 to 0.9 V in 0.1 M NaOH prior to their use as glucose sensors. Figure 5a shows the CV curves of a CuNi/C electrode in 60 cycles, at a scanning rate of 20 mV s^−1^. In the initial cycles, the active sites on the electrodes experience a conversion from Ni and Cu to Ni^3+^ and Cu^3+^ and then back to Ni and Cu, respectively, in the scan, and the process and their inverse processes can be expressed as shown in Eqs. 1–5. Equations 1, 3, and 4 correspond to the conversion of Cu and Ni to + 2 valency, and Eqs. 2 and 5 correspond to the conversion of + 2 to + 3 valency [48, 53, 54]. As shown in Fig. 5a, the peak currents corresponding to Eqs. 1, 3, and 4 decrease with the cycle number and even finally disappear while the peak currents corresponding to Eqs. 2 and 5 increase with increases in the cycle number and reach constant values. Therefore, the activation results in the active sites on electrode surface follow the reversible processes in Eqs. 2 and 5 by 60-cycle CVs. Additionally, Cu (III) and Ni (III) formed in the positive scan are used as catalysts to detect glucose as shown in Fig. 5b. The addition of glucose leads to an increase in the peak current due to the oxidation of glucose by Cu (III) and Ni (III) based on Eqs. 6 and 7 [48, 53]. For comparison purposes, the CV curves of Ni/C on Ni foam and raw Cu foam are shown in Fig. S6. When compared with those on the Ni/C, we concluded that the redox peaks on CuNi/C are hybrids of those on Ni and Cu that convert to high valence and turn back in the positive scan and subsequent negative scan.

The SEM images of activated CuNi/C electrodes (shown in Fig. S7) verify the formation of hydroxides that resemble pine needles (Fig. S7b) and evidently differs from the raw morphology (Fig. S7a). However, the needlelike hydroxides do not exhibit any interference with the array structure of raw CuNi/C electrode as shown in Fig. 3c, d. The Raman spectra of the activated electrodes (shown in Fig. S8) indicate the existence of NiOOH and CuOOH, and this is consistent with the CV results. The higher D peak when compared with G peak demonstrates that the carbon in the nanosheets is amorphous.

Activation of CuNi/C is as follows:

1 $${\text{Ni}} + 2 {\text{OH}}^{ - } \to {\text{Ni(OH)}}_{ 2} + 2 {\text{e}}^{ - }$$

2 $${\text{Ni(OH)}}_{ 2} + {\text{OH}}^{ - } \leftrightarrow {\text{NiOOH}} + {\text{H}}_{ 2} {\text{O}} + {\text{e}}^{ - }$$

3 $${\text{Cu}} + 2 {\text{OH}}^{ - } \to {\text{CuO}} + {\text{H}}_{ 2} {\text{O}} + 2 {\text{e}}^{ - }$$

4 $${\text{CuO}} + {\text{H}}_{ 2} {\text{O}} \to {\text{Cu(OH)}}_{ 2}$$

5 $${\text{Cu(OH)}}_{ 2} + {\text{OH}}^{ - } \leftrightarrow {\text{CuOOH}} + {\text{H}}_{ 2} {\text{O}} + {\text{e}}^{ - }$$

Glucose detection is as follows:

6 $${\text{N}}{\kern 1pt} {\text{iOOH}} + {\text{glucose}} \to {\text{Ni(OH)}}_{ 2} + {\text{gluconolactone}}$$

7 $${\text{CuOOH}} + {\text{glucose}} \to {\text{Cu(OH)}}_{ 2} + {\text{gluconolactone}}$$

The electrochemical characteristics of activated CuNi/C electrodes for detecting glucose were investigated. The CV curves of the electrodes in 0.1 M NaOH without and with 0.4 mM glucose are shown in Fig. S9a, b, respectively. The peak currents increase with increases in the scan rates in both the electrolytes, and the values in the glucose-containing electrolyte exceed those without glucose at the same scan rates. The additional currents are attributed to the oxidation of glucose [48, 53, 54], and this is verified by the CV curves of the electrode in 0.1 M NaOH with various concentrations of glucose at a constant scan rate as shown in Fig. S9c. The oxidizing current peak increases with increases in the glucose concentration, while the reduction current is steady due to the irreversible processes of glucose oxidation in Eqs. 6 and 7. Furthermore, the relationship between the peak current with the scan rates was examined and is shown in Fig. S9d. Both the anodic and cathodic peak currents are directly proportional to the square root of scan rates, and this implies a typical diffusion-controlled process.

**Fig. 6:**
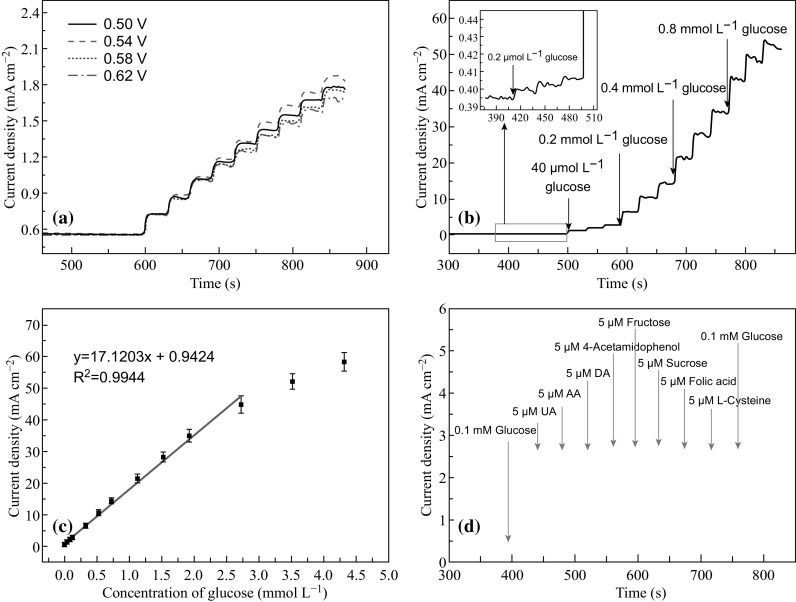
**a** Effects of various potentials on the amperometric response of CuNi/C electrodes given the successive addition of 8 μM glucose. **b** Amperometric responses of CuNi/C electrodes given the successive addition of glucose at 0.54 V. The left inset shows partial amplification of the amperometric response to low glucose concentration. **c** The corresponding calibration curve of the response current density relative to glucose concentration. **d** Interference test performed on CuNi/C electrodes by adding 0.1 mM glucose, 5 μM DA, 5 μM AA 52 μM UA, 5 μM acetaminophen, 5 μM fructose, 5 μM sucrose, 5 μM folic acid, and 5 μM l-cysteine into 0.1 M NaOH at 0.54 V

In order to select a reasonable potential for glucose detection, the amperometric responses of consecutively adding 8 μM glucose into 0.1 M NaOH at different potentials are investigated, and the results are shown in Fig. 6a. The detection potential should cater to the formation of high-valent Cu and Ni species as well as avoid oxygen evolution. Based on this, the potentials of 0.50, 0.54, 0.58, and 0.62 V are selected for comparison purposes in the present study. Evidently, the glucose oxidation at 0.54 V results in the maximum current response, and thus 0.54 V is set as the working potential in the subsequent investigation. The influence of pH value on the glucose determination was examined by the amperometric responses of CuNi/C electrodes given the successive addition of 20 μM glucose into NaOH solution with different concentrations at 0.54 V as shown in Fig. S10. It is observed that the response current is the highest and most stable in the solution of 0.1 M NaOH. In contrast, the response currents are negligible at lower pH values (e.g., pH = 7, 9, 11), and the response current fluctuates sharply at higher pH value (e.g., pH = 14). Therefore, 0.1 NaOH is used as the matrix solution of glucose detection.

The amperometric response to successive additions of glucose with specific concentrations into 0.1 M NaOH at 0.54 V was measured to study the detection limit, linear range, and sensitivity of CuNi/C electrode. The *i*–*t* curves are shown in Fig. 6b, and the inset shows the enlarged view of the responses for trace addition (0.2 µM). The detection limit is calculated based on the lowest added concentration that presents a clear response. In the present study, the detection limit is 0.067 µM with a signal to noise ratio of 3 (S/N = 3). The relationship that steady currents based on specific glucose concentrations (as shown in Fig. 6c) indicates a linear range from 0.2 μM to 2.72 mM (*R* ^2^ = 0.9953) and presents a sensitivity of 17.12 mA mM^−1^ cm^−2^. A comprehensive comparison between our CuNi/C electrodes and reported CuNi-based glucose sensors is shown in Table 1. The linear range of our electrode is slightly inferior. However, the reasonable detection limit and ultra-high sensitivity are its advantages when compared with previous electrodes. The CuNi/C electrodes possess the highest sensitivity (17.12 mM^−1^ cm^−2^), and this is at least an order higher than the other results. The linear range of CuNi/C (0.2–2720.6 µM) is slightly inferior when compared with those in extant studies. Nevertheless, it considerably exceeds those of the pristine Ni/C electrodes (approximately 0.15–1480 µM) reported in a previous study [43].

**Table 1:** Comparison of CuNi/C glucose sensor with previously reported Ni-based non-enzymatic glucose sensors

Electrode composition	Working potential (V vs. Ag/AgCl)	Sensitivity mA (mM^−1^ cm^−2^)	Linear range (µM)	Detection limit (µM)	Self-supported or not
CuNi/MWCNTs [38]	0.450	1.4702	0.962–5000	0.0025	No
CuNi/MWCNTs [37]	0.575	2.4370	2000–8000	0.0250	Yes
CuNi [35]	0.550	0.01916	7–23670	2.3000	Yes
CuNi [36]	0.600	1.5909	10–3200	5.0	Yes
This work	0.540	17.1203	0.2–2720.6	0.06667	Yes

The selectivity of CuNi/C electrode in glucose detection was investigated by introducing AA, DA, UA, acetaminophen, fructose, sucrose, folic acid, and l-cysteine into electrolytes to inspect its ability to discriminate between interference species. Figure 6d shows the amperometric responses of CuNi/C electrode toward the electrolyte while adding 5 μM AA, 5 μM DA, 5 μM UA, 5 μM acetaminophen, 5 μM fructose, 5 μM sucrose, 5 μM folic acid, 5 μM l-cysteine, and 0.1 mM glucose into 0.1 M NaOH solution at 0.54 V. Evidently, the jamming signals from the interferents are almost negligible when compared with the response to glucose. The same response currents of CuNi/C to glucose between the initial 0.1 mM addition and the final addition after the interfering species suggest good reliability. Furthermore, the feasibility of CuNi/C in physiological environments was evaluated by the tolerance of chloride poisoning. The almost coincident curve patterns of CVs (Fig. S11) and amperometric responses (inset of Fig. S11) demonstrate the high tolerance of CuNi/C toward chloride ions.

Three significant properties, namely, reusability, reproducibility, and stability of CuNi/C were investigated by inspecting amperometric responses in various situations as shown in Fig. S12. A CuNi/C was used to detect the reusability of the addition of 0.2 mM glucose five times as shown in Fig. S12a. The low relative standard deviation (RSD) approximately 2.07% of the five response currents reveals the good reusability of CuNi/C for glucose sensing. Similarly, the same analysis was performed on five electrodes to inspect the reproducibility of CuNi/C as shown in Fig. S12b. The fair RSD approximately 3.01% of the five response currents suggests good consistency of the electrodes. The stability of CuNi/C electrodes was inspected by testing the current response to 8 μM glucose every 5 days in the 60-day period as shown in Fig. S12c. The response currents of the electrode retain 90% of the initial value through 60 days, and this reveals its excellent long-term stability. The irregular degradation of response current is ascribed to the room temperature variation. Although the electrode process is sensitive to temperature, we continue to conduct measurements without a thermostat, to imitate real operating conditions. However, the current density decay does not exceed 8% in the long stability test. The attractive merits of reusability, reproducibility, and stability suggest that CuNi/C electrodes are a good alternative for practical glucose detection.

In order to further verify its practicality, human blood serum was tested by a CuNi/C electrode by using amperometric response. The serum (30 μL) obtained from a hospital without any further treatment was added into 0.1 M NaOH solution (10 mL), and the sample was measured using a potentiostatic method at 0.54 V (vs. Ag/AgCl) in a three-electrode cell (a Pt foil as counter electrode). The glucose concentrations of serum are obtained by measuring their response currents in NaOH matrix solution, and thus it is unnecessary to test their CVs although amperometric responses are recorded. Conversely, the CV measurement is not conducted on the as-prepared serum samples, and amperometric responses are tested for sustaining less than 30 s. Figure S13 shows the amperometric response (at 0.54 V) of serum sample 1 for a test. As shown in Table 2, our results are in agreement with the results obtained by clinical reports (with respect to the method of glucose-6-phosphate dehydrogenase) in hospitals. The recovery assessed by standard additions of glucose to the serum samples was close to 100%, and this implies that the CuNi/C sensors are promising in terms of glucose detection with appealing accuracy.

**Table 2:** Adding standard recovery results to determine glucose in blood serum samples

Sample	Hospital results^a^ (mM)	Our results (uncertainty) (mM)	RSD (%)	Added amount (mM)	Our results (uncertainty) (mM)	Recovery (%)	RSD (%)
1	6.050	6.160 (0.12)	1.16	4.0	10.32 (0.15)	106.7	3.91
2	8.190	7.970 (0.18)	2.35	6.0	14.40 (0.14)	103.5	
3	15.77	15.61 (0.17)	2.61	8.0	23.69 (0.16)	98.96	

^a^The standard uncertainty of glucose-6-phosphate dehydrogenase method to detect glucose is 0.11–0.18

## Conclusion

In this study, CuNi/C nanosheet arrays on Ni foam are prepared by electrodepositing Cu on a Ni-MOF derivate. Physical measurements results indicate that Cu nanoparticles are homogeneously distributed on the Ni/C nanosheets without interference to the array structure. The CuNi/C self-supported electrodes are applied as electrochemical sensors to detect glucose. The electrochemical results demonstrate that the electrodes possess a detection limit of 0.067 µM, a linear range from 0.2 μM to 2.72 mM, and a sensitivity of 17.12 mA mM^−1^ cm^−2^, and that their behavior is better than that of previous Ni/C electrodes [43]. The tolerance of CuNi/C toward AA, DA, UA, and chloride ions reveals its good selectivity and resistance to poison. The most important advantages of this sensor include its good reusability, reproducibility, and stability given the controllable preparation of electrodes and the stable chemical state on their surface. The detection of glucose in human blood serum presents results similar to those obtained from the method of glucose-6-phosphate dehydrogenase. All the results indicate that the prepared CuNi/C electrodes are good alternatives for non-enzymatic sensors of glucose detection.

## Electronic supplementary material

Below is the link to the electronic supplementary material.

Supplementary material 1 (PDF 1420 kb)
