# Supplementary material for: A CuNi/C Nanosheet Array Based on a Metal–Organic Framework Derivate as a Supersensitive Non-Enzymatic Glucose Sensor
Source: Nanomicro Lett. 2017 Dec 22;10(2):28. doi: 10.1007/s40820-017-0178-9 (PMC6199074; doi:10.1007/s40820-017-0178-9)
Supplement: Supplementary file 1 — Supplementary material 1 (PDF 1420 kb) [file 40820_2017_178_MOESM1_ESM.pdf]

Supporting Information for

A CuNi/C Nanosheet Array Based on Metal-Organic Framework Derivate as a Supersensitive Non-Enzymatic Glucose Sensor

Li Zhang[1], Chen Ye[1], Xu Li[2], Yaru Ding[1], Hongbo Liang[1], Guangyu Zhao[1], Yan Wang[1,*]

[1]School of Chemistry and Chemical Engineering, Harbin Institute of Technology, Harbin, Heilongjiang 150080, People's Republic of China

[2]Department of Ophthalmology, the Second Hospital, Jilin University, Changchun, Jilin 130022, People's Republic of China

*Corresponding author. E-mail: wangy_msn@hit.edu.cn

Tel/Fax: +86 451 86403719

Figures

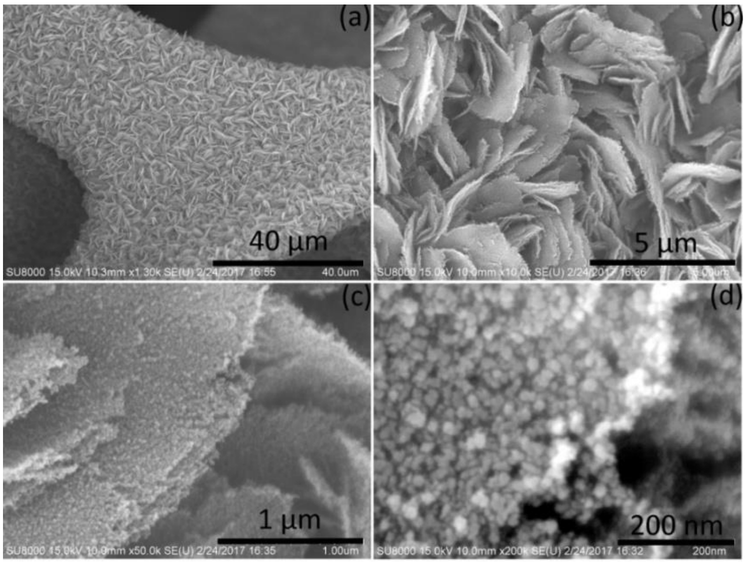

Fig. S1 SEM images of Ni/C nanosheet arrays derived from Ni-MOFs

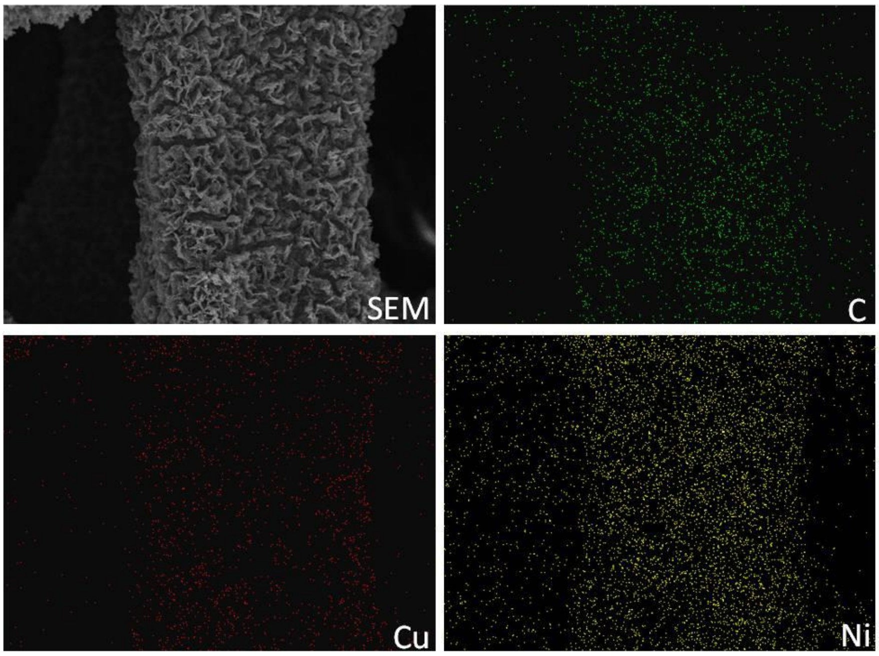

Fig. S2 Element mapping from EDS test

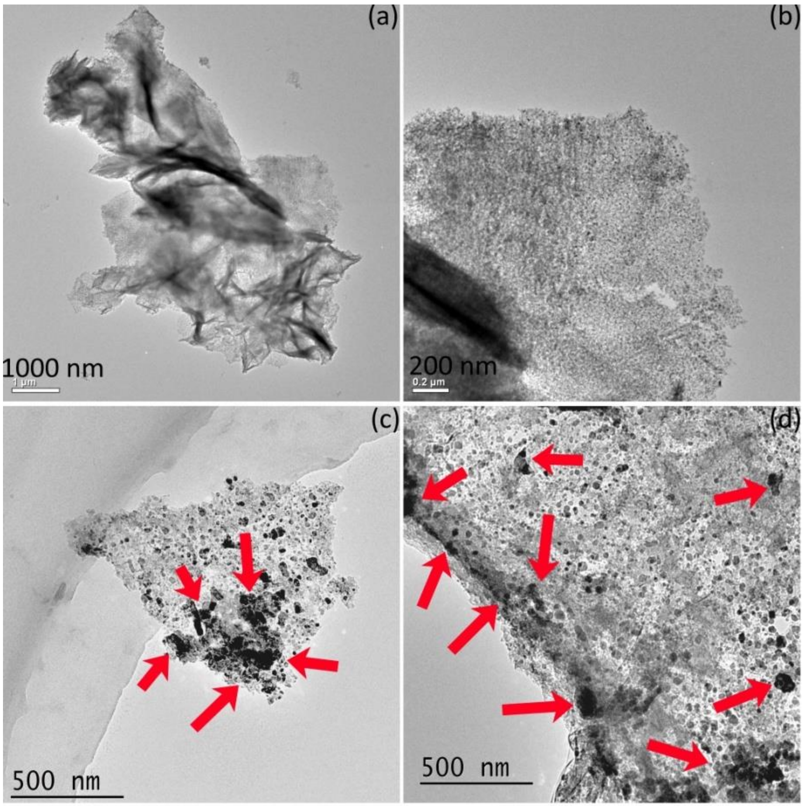

Fig. S3 TEM images of **a, b** Ni/C and **c, d** CuNi/C nanosheets. Cu nanoparticles are pointed by red arrows.....

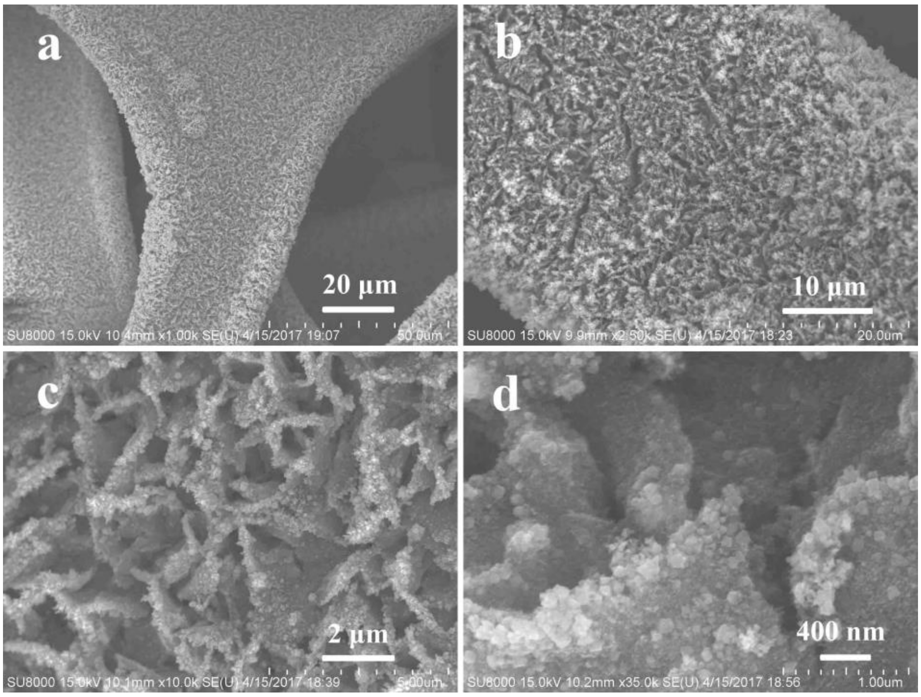

Fig. S4 SEM images of CuNi/C electrodes prepared by depositing Cu with 20 s

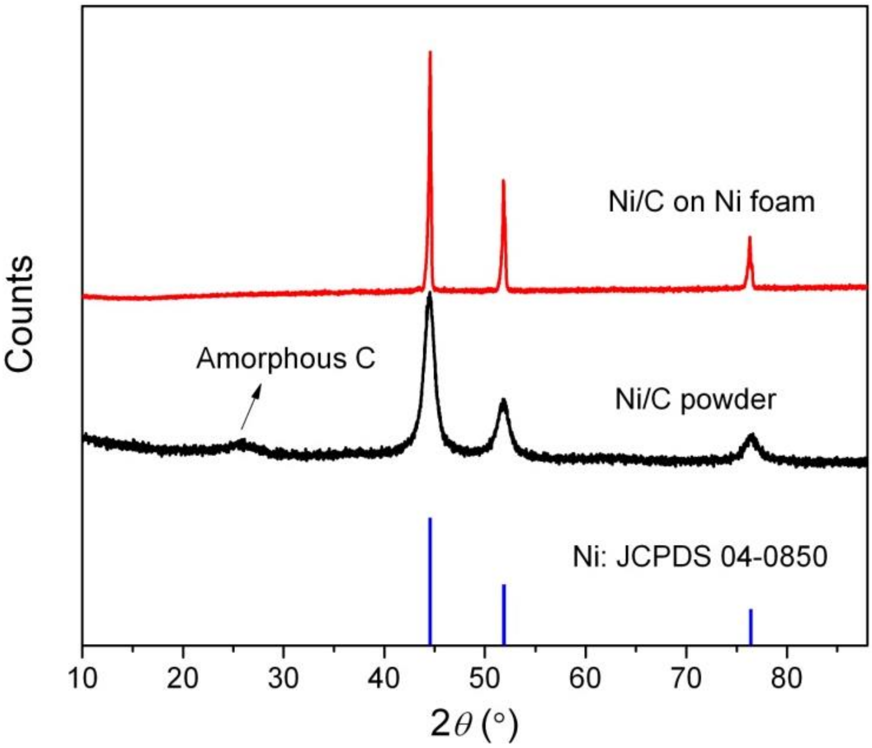

Fig. S5 XRD patterns of Ni/C on Ni foam and Ni/C powder

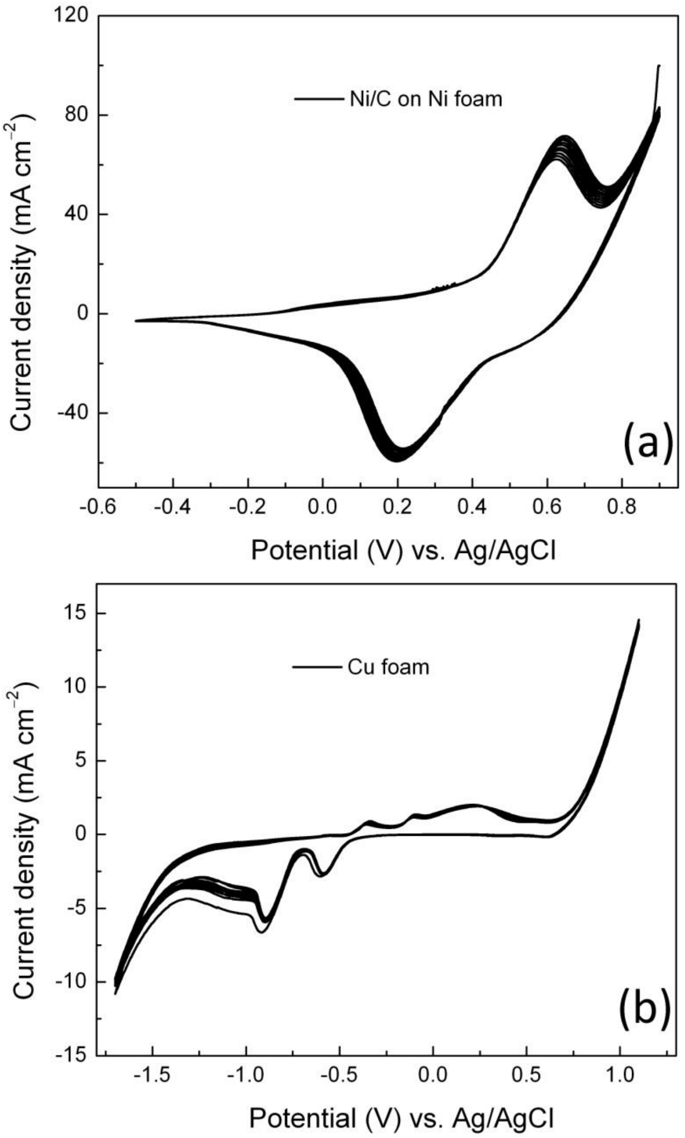

Fig. S6 CV curves of **a** Ni/C on Ni foam and **b** raw Cu foam in 0.1 M NaOH. The CV measurements were carried out in a three-electrode system using Pt foil as counter electrode, an Ag/AgCl as reference electrode. The scan rate is 20 mV s^{-1}.

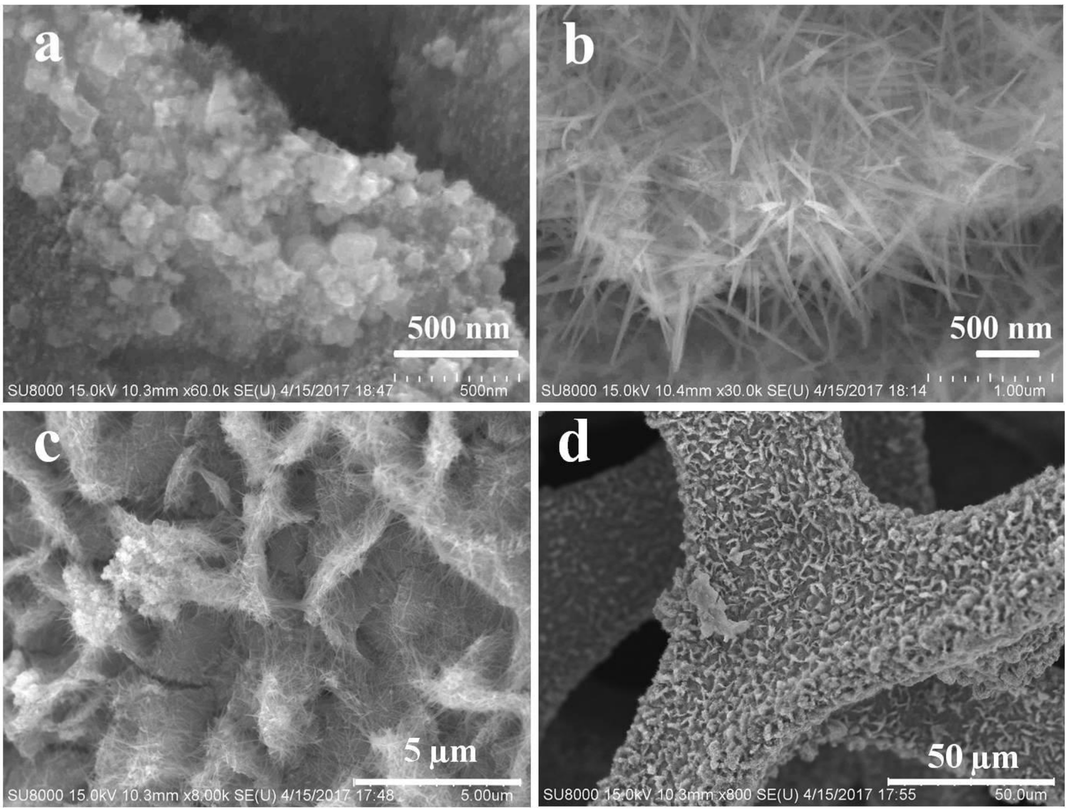

Fig. S7 SEM images of CuNi/C electrode **a** before and **b, c, d** after activation

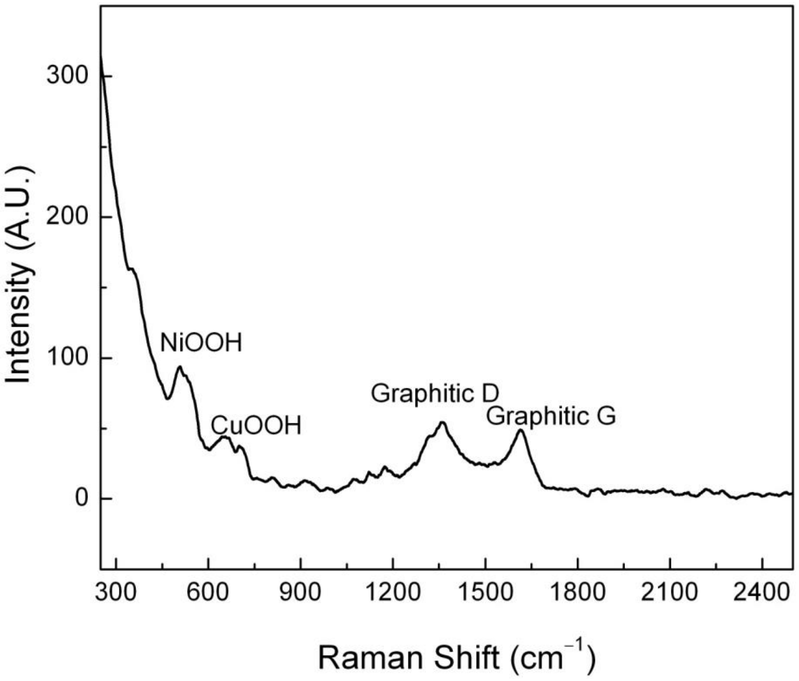

Fig. S8 Raman spectra of activated CuNi/C electrodes

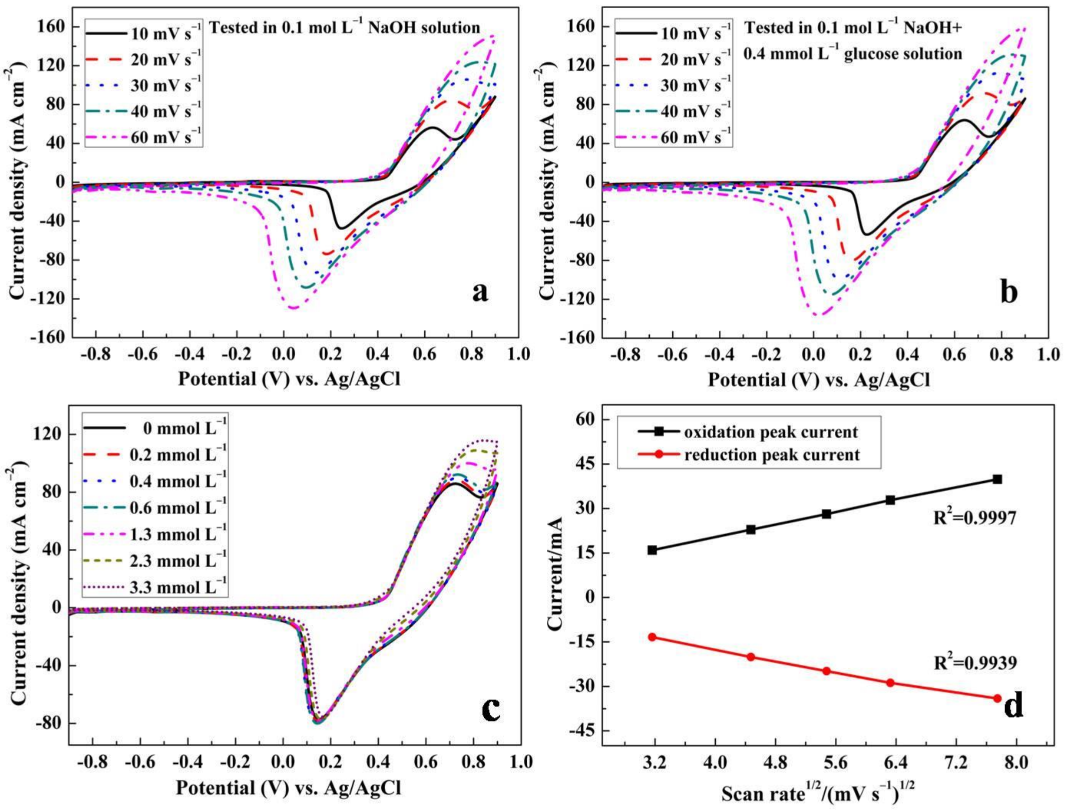

Fig. S9 CV curves of activated CuNi/C electrodes with various scan rates in **a** 0.1 M NaOH and **b** 0.1 M NaOH + 0.4 mM glucose. **c** CV curves in 0.1 M NaOH with various concentrations of glucose at a scan rate of 20 mV s^{-1}. **d** Linear plots of peak currents versus the square root of the scan rates

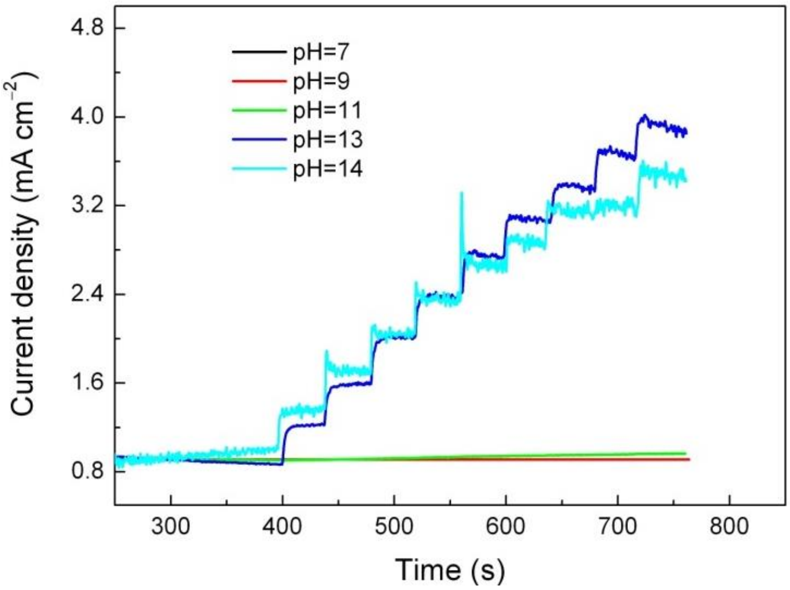

Fig. S10 Amperometric responses of CuNi/C on the consecutive addition of 20 μM glucose into NaOH solution with different concentrations at 0.54 V

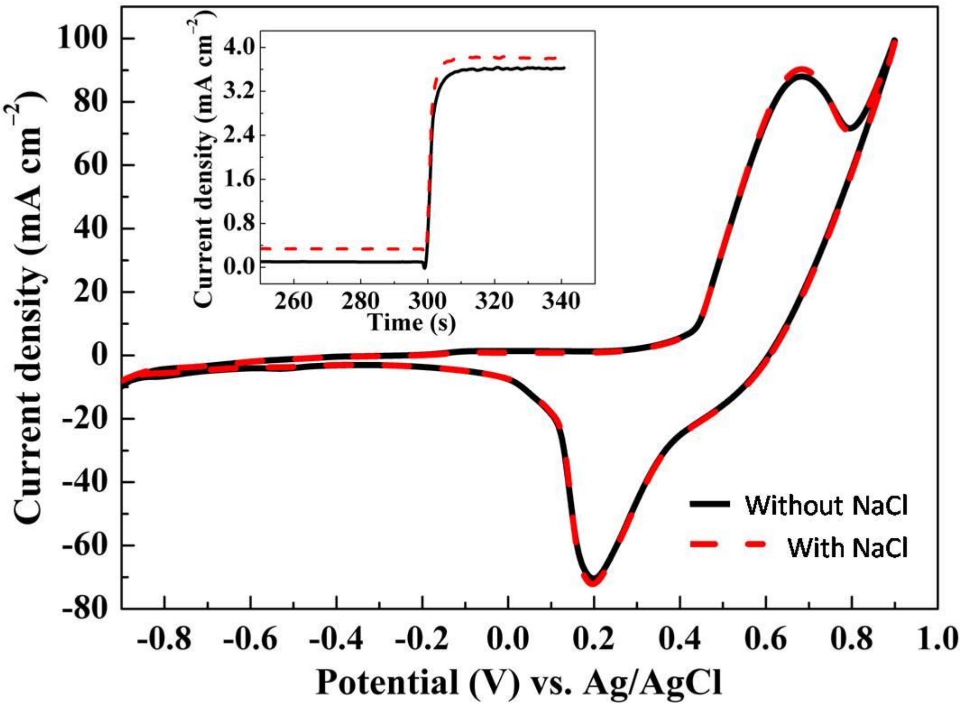

Fig. S11 CVs of the CuNi/C electrodes containing 0.2 mM glucose with and without 20 mM NaCl in 0.1 M NaOH solution, the inset is the corresponding amperometric responses

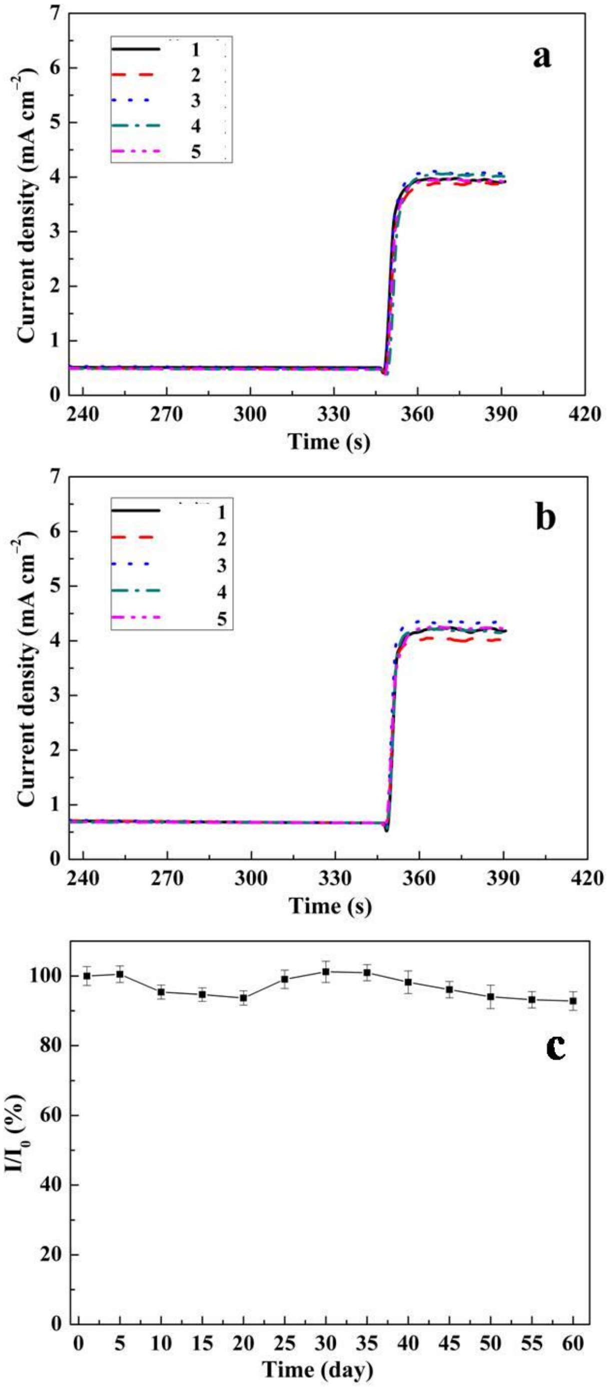

Fig. S12 a Amperometric responses of an electrode to the addition of 0.2 mM glucose solution for respective five times. **b** Amperometric responses of five electrodes fabricated on the same condition to the addition of 0.2 mM glucose solution. **c** Long-term stability test of CuNi/C electrodes stored at room temperature over a 60-day period

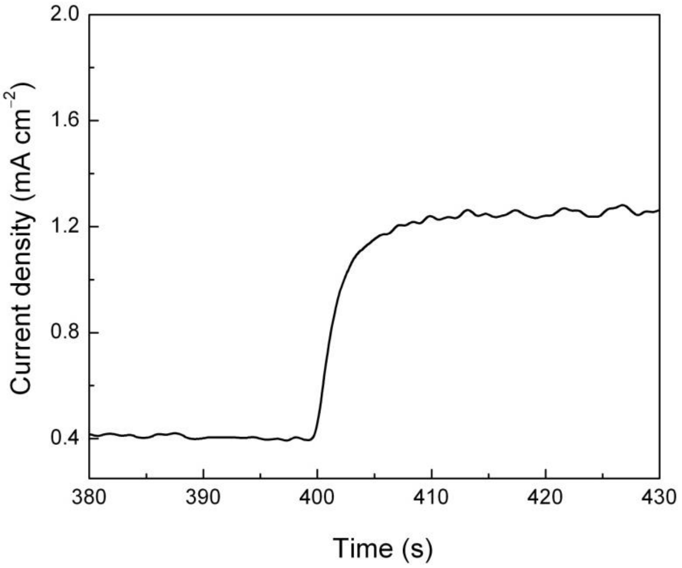

Fig. S13 Amperometric response of CuNi/C electrodes upon serum sample 1 at 0.54V
